# The Free Caesareans Policy in Low-Income Settings: An Interrupted Time Series Analysis in Mali (2003–2012)

**DOI:** 10.1371/journal.pone.0105130

**Published:** 2014-08-19

**Authors:** Pierre Fournier, Alexandre Dumont, Caroline Tourigny, Aline Philibert, Aliou Coulibaly, Mamadou Traoré

**Affiliations:** 1 Research Center of the University of Montreal Hospital (CR-CHUM), Montreal, Canada; 2 School of Public Health, University of Montreal, Montreal, Canada; 3 Research Institute for Development, Université Paris Descartes, Sorbonne Paris Cité, UMR 216, Paris, France; 4 Unité de recherche et de formation en santé de la mère et de l'enfant (URFOSAME), Referral health center of the Commune V, Bamako, Mali; Iran University of Medical Sciences, Islamic Republic of Iran

## Abstract

**Introduction:**

Several countries have instituted fee exemptions for caesareans to reduce maternal and newborn mortality.

**Objectives:**

To evaluate the effect of fee exemptions for caesareans on population caesarean rates taking into account different levels of accessibility.

**Methods:**

The observation period was from January 2003 to May 2012 in one Region and covered 11.7 million person-years. Exemption fees for caesareans were adopted on June 26, 2005. Data were obtained from a registration system implemented in 2003 that tracks all obstetrical emergencies and interventions including caesareans. The pre-intervention period was 30 months and the post-intervention period was 83 months. We used an interrupted time series to evaluate the trend before and after the policy adoption and the overall tendency.

**Findings:**

During the study period, the caesarean rate increased from 0.25 to 1.5% for the entire population. For women living in cities with district hospitals that provided caesareans, the rate increased from 1.7% before the policy was enforced to 5.7% 83 months later. No significant change in trends was observed among women living in villages with a healthcare centre or those in villages with no healthcare facility. For the latter, the caesarean rate increased from 0.4 to 1%.

**Conclusions:**

After nine years of implementation policy in Mali, the caesarean rate achieved in cities with a district hospital reached the full beneficial effect of this measure, whereas for women living elsewhere this policy did not increase the caesarean rate to a level that could contribute effectively to reduce their risk of maternal death. Only universal access to this essential intervention could reduce the inequities and increase the effectiveness of this policy.

## Introduction

Maternal mortality is a major health problem. Although significant progress has been made at the global level, progress in Africa, and in specific countries such as Mali, has been inadequate [1], [2]. Effective solutions are known, such as intra-partum care strategies including emergency obstetrical care (EmOC) [3]. However, these are difficult to implement in low-income countries because of numerous obstacles related to availability, quality, geographic accessibility and funding [4].

In settings with poor EmOC coverage, caesareans can improve the maternal and neonatal outcomes if used in a timely manner for women who really need them [5]. During the 1990s, the rate of caesareans increased in developing and emerging countries, with the exception of sub-Saharan Africa, where it was estimated to be 2.9% between 1990 and 2003 [6]. There were also significant intra-country disparities, with women from the poorest households and those from rural settings much less likely to deliver by caesarean [7].

There is no optimal population caesarean rate. Rather, there is a minimum threshold, considered to be between 1% and 3% according to some authors [8], [9] but determined as 5% by the World Health Organization [10]. Above 15%, there is a negative impact on both mothers and newborns [11]. A maternal mortality reduction strategy based only on caesareans has limitations, especially in Africa where caesareans are often carried out in emergency situations, which inherently do not improve maternal and perinatal outcomes. The Latin American experience shows that when caesarean rates exceed 15% to 20%, non-justified caesareans lead to higher maternal-infant mortality rates [12].

There are many barriers to access to EmOC, including costs. Several countries have taken important measures focused on making caesareans free [13]. These interventions have been implemented in fragile and inequitable healthcare systems, raising the question of whether these disparities might not limit their effectiveness [14].

## Context

Mali is a large African country of 1.2 million km^2^, 14.5 million inhabitants (2009) [15] and ranked 182^th^ out of 186 on the Human Development Index [16]. Maternal health is a major issue, and maternalmortality ratio is 550 maternal deaths per 100 000 living births (range if uncertainty between 330 and 940 maternal deaths) [17]. A significant proportion of deliveries occur at home; estimates for 2002–2005 were 19% in urban and 65% in rural settings [18]. EmOC coverage is also low.

Mali has made significant efforts to improve its healthcare system and has adopted numerous reforms [19]. To reduce maternal and neonatal mortality and to facilitate access to EmOC, two major programs were implemented. Since 2000, a referral system (Ref-Syst) was gradually set up in the country's 57 districts. It includes a component of improved EmOC, an ambulance network, and a community-based fund [20]. In 2005, fee exemptions for caesareans were instituted throughout the country. This measure (Free-CSec), covers hospital costs (hospitalisation, drugs, supplies and professional fees). While this measure eliminated some significant financial barriers, women living far from points of service still had to pay for transportation and other medical costs [21].

The Kayes health region covers a broad territory (120,000 km^2^) with 2 million people (2009) [15] living in seven districts. Between 2000 and 2005 the Ref-Syst was implemented in the seven districts, leading to increased access to referral hospitals and improving maternal and perinatal outcomes [20]. Another study showed that, even with Ref-Syst, the transportation time increased lethality and diminished the benefits of caesareans [22].

In Mali, as in other countries that have opted for universal fee exemptions for emergency obstetrics, it is legitimate to question their effectiveness because these policies are costly and resources are scarce. But as these policies are universal, experimental studies are not possible. Therefore, rigorous observational studies must be used to shed as much light as possible on these policies [23]. Platforms for monitoring maternal and newborn outcomes can replace experimental designs [24].

## Objectives

This study aimed to analyse the trends in population caesarean rates pre and post Free-CSec using interrupted time series. As the pre Free-CSec period includes sub periods with and without Ref-Syst, we also assessed the influence of Ref-Syst and the area of residence on caesarean rates.

## Methodology

### Population

Of the seven districts of the Kayes region, two were not included in this study. The first was the Kayes district, where the regional hospital is located. The mainly urban population of the Kayes district does not encounter the same problems of geographic access and financial constraints that arise in the other districts, whose populations are mostly rural. In that district there are specialists (obstetricians, anesthesiologists, and pediatricians), whereas in district hospitals caesareans are done by general practitioners with surgical training. The second district excluded was Kenieba, where the Ref-Syst was implemented after the Free-CSec policy.

To determine whether caesarean rates varied according to area of residence, we considered three zones: (1) cities with district hospitals; (2) villages with primary healthcare centres; (3) villages without a healthcare facility. In addition to providing an approximate correspondence to geographic accessibility, these zones represent different levels of organisational accessibility. In (1), services are directly accessible. In (2), the Ref-Syst facilitates transportation (ambulance availability) and covers part of the costs of transportation and services, although it is not guaranteed to be always fully operational. For (3), women and their families must first find transportation to go to the primary healthcare centre or directly to the district hospital, which entails travel over distances up to 100 km and involves considerable effort and cost.

### Study periods

The policy of fee exemptions for caesareans was announced on June 26 2005. To evaluate its effects, we used a time series beginning on January 2003 and ending in May 2012. This series includes a pre-intervention period of 30 months (January 1^st^ 2003 to June 30^th^ 2005) and a post intervention period of 83 months (July 1^st^ 2005 to May 31^st^ 2012).

### Outcome and data

The assessed outcome is the estimated monthly caesarean rate (number of caesarean deliveries/total number of deliveries). The numerator is the number of caesarean cases identified from the system GESYRE (*Gestion du Système de Référence Evacuation*) and the denominator is the estimate of the total number of births. GESYRE, implemented in 2003 as part of a collaborative research program with the health and social authorities of the Kayes region, is a registration system for all obstetrical emergencies and interventions including caesareans [25]. The denominator was determined using data from the 1998 and 2009 censuses at the village level. The total number of births for each commune was estimated from the 1998 and 2009 censuses and the annual crude birth rates as follows: Population from the 69 communes included in the study was estimated from 2003 to 2012 using their specific growth rate observed between the two censuses. The expected number of deliveries in one specific commune (a) is the population in year (i) x crude birth rate for year (i). (For more details see Text S1: Population and deliveries estimates).

Thus, the population caesarean rate for a given geographic area and time period is the number of caesareans carried out in that period among women living in that geographic area divided by the number of births expected in that area for that period.

### Statistical analysis

Healthcare policies that are applied to the population at large inherently cannot be studied using experimental designs, and interrupted times series are appropriate alternatives to randomised trials [26]. Their use is recommended for clinical evaluations [27] and for measuring the effects of public policies on health [28]–[29].

Since monthly data were collected over time they were suitable for interrupted time series analysis. We used segmented linear regression models to estimate the change in the caesarean rate before and after the Free-CSec policy, immediately and over time. The Durbinalt test, a Durbin-Watson's alternative test for serial correlation in data, showed a moderate serial autocorrelation, such as the partial autocorrelation plot. Failing to correct for autocorrelation in longitudinal data may lead to underestimated standard errors and overestimated significance of the effects of an intervention. Therefore we used an extended ordinary least-squares (OLS) regression model divided into pre and post-intervention segments while adjusting the variance estimation by the Newey-West standard errors method that corrected for serial correlation in residuals. The maximum lag to be considered in the autocorrelation structure was determined by visual inspection and with confidence intervals calculated using a standard error of 1/sqrt(n).

The segmented regression model best fits an OLS regression line as follows [30]: 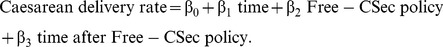



β_0_ estimates the baseline caesarean rate at the beginning of the pre Free-CSec period.

β_1_ estimates the change in rate that occurs with each month before the Free-CSec policy.

β_2_ estimates the change in the caesarean rate immediately after the Free-CSec policy.

β_3_ estimates the change in the trend of the rate of the post-Free-CSec period compared to the pre-Free-CSec period.

To describe a clinically meaningful absolute reduction, the absolute effect (β2+β3 * Number of months after intervention) was estimated by difference between the estimated outcome at a certain time after the intervention and the outcome at that time if the intervention not taken place Its standard error was calculated including the covariance of level and slope terms [30]–[31].

The statistical significance for parameter estimation was set at α = 5% (p≤0.05).

All analyses were performed using Stata 11 [32].

### Ethics Statement

This research was approved by the Ethics Committees of the University of Montreal Hospital Research Centre (Canada) and the Faculty of Medicine, Pharmacy and Odonto-Stomatology of the University of Bamako (Mali). No written consent was obtained from participants for using their clinical records, but patient records/information was anonymized and de-identified prior to analysis.

## Results

Table 1 presents the population and services data for the pre and post Free-CSec policy periods.

**Table 1:** Population and service data before and after fee exemptions for caesareans (Free-CSec).

	Before Free-CSec	After Free-CSec
	Jan 2003 – June 2005	July 2005 – May 2012
**Population data**		
Population (persons-year)^a^	2 759 572	8,973 483
Expected deliveries		
Cities with district hospital	11,390	36,362
Villages with health centre	29,021	124,330
Villages with no health care facility	94,231	261,913
Total	134,642	422,605
**Service data**		
Facilities-based deliveries numbers and (rates)^b^	34,455 (25.59%)	248,968 (58.91%)
Caesareans	599	4,725
Caesareans (facilities rate)^c^	1.74%	1.90%
Caesareans (population rate)^d^	0.44%	1.12%

a See Text S1.

b Observed deliveries/Expected deliveries.

c Observed caesarean sections/Observed deliveries.

d Observed caesarean sections/Expected deliveries.

We registered 5375 women during the study period, 51 (0.9%) were excluded as their health area and village were unknown. For 28 women (0.5%), their health area was known, but their village was missing: we allocated them in the most frequent category in their respective year. Over the time covered by this study, the rates of facility-based and caesarean deliveries increased considerably after the Free-CSec policy: respectively from 26% before to 60% after (2.7 fold) and from 0.44% to 1.22% (2.5 fold).

**Figure 1:**
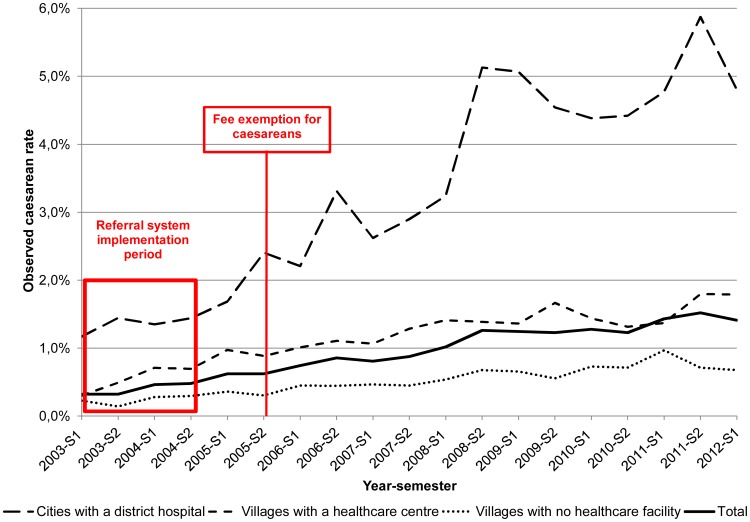
Population caesarean rates by semester and area of residence (January 2003-May 2012).

There was an increase of the caesarean rates for all the periods, after and before the Free-CSec policy and in all the strata, with a stronger trend for the cities with hospitals strata (Figure 1).

**Figure 2:**
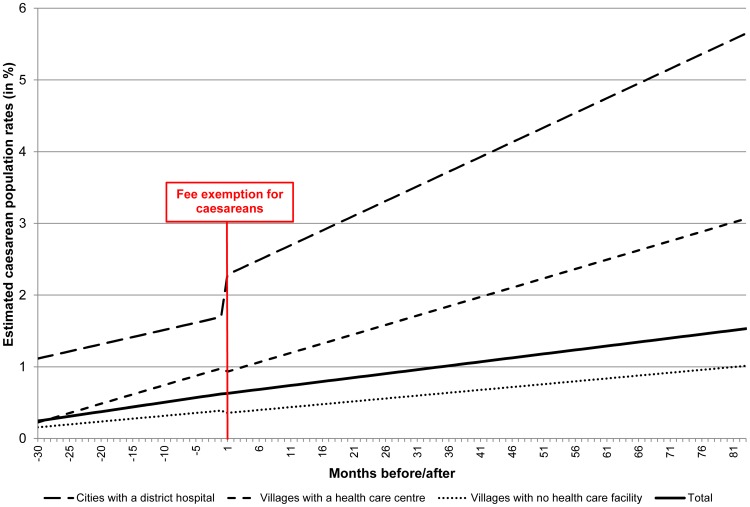
Fitted lines after-before the fee exemptions for caesareans (January 2003-May 2012).

Figure 2 shows the fitted lines for the pre and post Free-CSec period and Table 2 provides the parameter estimates for these interrupted time series. For example, the value of the parameter β1 for the total population is 0.013 and its p value is <0.001. This means that the slope of the curve (meaning the trend during the pre intervention) period was positive (the trend is an annual increase) and that trend was statistically significant. In contrast, after the intervention the difference between the slopes of the curves before and after the intervention was negative (β3 = −0.002) and this was not statistically significant (p = 0.305). The caesarean rates increased of 2.29% in cities with district hospitals while it decreased of 1.50% in villages with no healthcare facilities.

**Table 2:** Parameter estimates from the segmented regression model based on Newey-West method for the Free-CSec policy (January 2003 to May 2012).

Areas of residence	Coefficient	S.E.	*t*-statistic	p-value	95% CI
**Villages with no healthcare facility**					
*Fit test (Wald test)* a	*F = 53.12; p<0.001*				
Intercept (β0)	0.157	0.038	4.17	<0.001	(0.08; 0.23)
Trend before the intervention (β1)	0.008	0.002	4.29	<0.001	(0.00; 0.01)
Change right after the intervention (β2)	−0.037	0.044	−0.85	0.398	(−0.12; 0.05)
Trend change after the intervention (β3)	−0.002	0.002	−0.91	0.366	(−0.01; 0.00)
Absolute effect (S.E)	−0.203	0.041			
**Villages with a healthcare centre**					
*Fit test (Wald test)* a	*F = 160.24; p<0.001*				
Intercept (β0)	0.225	0.049	4.59	<0.001	(0.13; 0.32)
Trend before the intervention (β1)	0.026	0.004	6.13	<0.001	(0.02; 0.03)
Change right after the intervention (β2)	−0.088	0.110	−0.80	0.426	(−0.30; 0.13)
Trend change after the intervention (β3)	−0.017	0.004	−3.80	<0.001^CT^	(−0.03; −0.01)
Absolute effect (S.E)	−1.499	2.247			
**Cities with district hospital**					
*Fit test (Wald test)* a	*F = 95.65; p <0.001*				
Intercept (β0)	1.115	0.115	9.67	<0.001	(0.89; 1.34)
Trend before the intervention (β1)	0.020	0.008	2.44	0.016	(0.00; −0.04)
Change right after the intervention (β2)	0.550	0.293	1.88	0.063	(−0.03; 1.13)
Trend change after the intervention (β3)	0.021	0.009	2.48	0.015^CT^	(0.00; 0.04)
Absolute effect (S.E)	2.293	5.258			
**Total**					
*Fit test (Wald test)* a	*F = 234.26; p<0.001*				
Intercept (β0)	0.245	0.331	7.41	<0.001	(0.18; 0.31)
Trend before the intervention (β1)	0.013	0.002	5.79	<0.001	(0.09; 0.01)
Change right after the intervention (β2)	−0.003	0.062	−0.05	0.963	(−0.01; 0.12)
Trend change after the intervention (β3)	−0.002	0.002	−1.03	0.305	(−0.01; 0.00)
Absolute effect (S.E)	−0.169	0.029			

a Wald test of simple and composite linear hypotheses.

CT Significant change in trend after the intervention.

**Figure 3:**
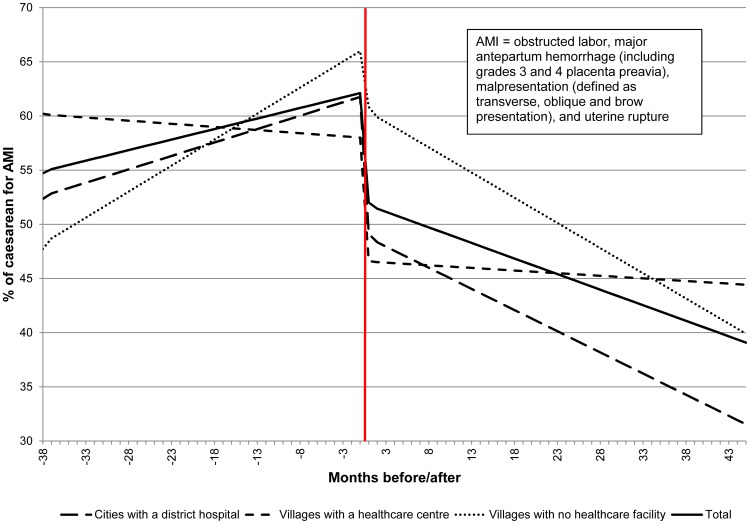
Fitted lines for the proportion of the caesarean for Absolute Maternal Indication (AMI) during the post Free-CSec period (July 2005-May 2012).

Figure 3 shows the fitted lines for the proportion of the caesarean sections for the Absolute Maternal Indication (AMI) during the post Free-CSec period. This period was divided into two parts: after and before September 1^st^ 2008 (38 months before and 44 months after). Table 3 provides the parameter estimates for these interrupted time series. After September 1^st^ 2008, the proportion of caesareans for AMI decreased of 85% in villages with no health care facilities and 65% in cities with district hospitals. The diminution for villages with a health care centre did not exceed 10%.

**Table 3:** Parameter estimates from the segmented regression model based on Newey-West method Caesareans for Absolute Maternal Indications (AMI) in post Free CSec Policy (July 2005–May 2012).

Areas of residence	Coefficient	S.E.	*t*-statistic	p-value	95% CI
**Villages with no healthcare facility**					
*Fit test (Wald test)* a	*F = 20.36; p<0.001*				
Intercept (β0)	47.736	3.908	12.21	<0.001	(39.96;55.52)
Trend before the intervention (β1)	0.480	0.149	3.22	0.002	(0.18;0.78)
Change right after the intervention (β2)	−5.159	3.559	−1.45	0.151	(−12.24;1.92)
Trend change after the intervention (β3)	−0.945	0.211	−4.48	<0.001^CT^	(−1.36;−0.53)
Absolute effect (S.E)	−83.594	2184.534			
**Villages with a healthcare centre**					
*Fit test (Wald test)* a	*F = 26.98; p<0.001*				
Intercept (β0)	60.209	3.476	17.32	<0.001	(53.29;67.13)
Trend before the intervention (β1)	−0.058	0.141	−0.41	0.682	(−0.34;0.22)
Change right after the intervention (β2)	−11.398	4.039	−2.82	0.006^CA^	(−19.44;−3.36)
Trend change after the intervention (β3)	0.009	0.139	0.07	0.946	(−0.27;0.29)
Absolute effect (S.E)	−10.651	121.044			
**Cities with a district hospital**					
*Fit test (Wald test)* a	*F = 33.24; p<0.001*				
Intercept (β0)	52.354	2.701	19.38	<0.001	(46.98;57.73)
Trend before the intervention (β1)	0.248	0.162	1.53	0.131	(−0.08;0.57)
Change right after the intervention (β2)	−12.635	5.355	−2.36	0.021^CA^	(−23.29;−1.98)
Trend change after the intervention (β3)	−0.638	0.162	−3.94	<0.001^CT^	(−0.96;−0.32)
Absolute effect (S.E)	−65.589	1657.060			
**Total**					
*Fit test (Wald test)* a	*F = 78.01; p<0.001*				
Intercept (β0)	54.708	2.121	25.80	<0.001	(50.49;58.93)
Trend before the intervention (β1)	0.195	0.078	2.49	0.015	(0.04;0.35)
Change right after the intervention (β2)	−10.090	1.704	−5.92	<0.001^CA^	(−13.48;−6.67)
Trend change after the intervention (β3)	−0.482	0.093	−5.16	<0.001^CT^	(−0.67;−0.26)
Absolute effect (S.E)	−50.096	979.564			

a Wald test of simple and composite linear hypotheses.

CA Significant change right after the intervention.

CT Significant change in trend after the intervention.

In January 2003, the population-based caesarean rate was 0.25% (estimates from the interrupted time series) with considerable differences between the residence areas: 1.12% for women living in cities with a hospital as compared to 0.16% for those living in villages without any health facilities. During the pre-intervention period, these rates rose respectively to 0.62%, 1.70% and 0.40%. All the slopes (β1) were positive and statistically different from zero, indicating an increasing trend of the caesarean rate. After the Free-CSec policy the picture changed dramatically. First, the trend (β3-slope change) for the population as a whole did not change significantly; the only noticeable changes were a significant increase for the women living in cities with a hospital and a significant decrease for those living in the villages with a health centre. At the end of the study period the caesarean rates reached 1.53% for the general population, 5.65% for the women living in cities with a hospital, 3.01% for those in the health centre villages and 1.02% for those living in villages without any health facilities.

As the proportion of caesarean rates for AMI showed an increase during the first years after the Free-CSec and a decrease later, a separate analysis on caesarean rates for AMI was undertaken for this period. The inflection point was set at September 1^st^ 2008, giving periods of 38 months before and 45 months after. In the before period, the proportion of caesareans for AMI for the total population rose from 55 to 64% and then fell dramatically from 53 to 38%.

As the pre Free-CSec period includes sub periods with and without Ref-Syst, we undertook a similar analysis (see Figure S1: Interrupted times series: after-before referral system; Figure S2: Fitted lines after-before referral system; Table S1: Parameter estimates from the segmented regression model for the referral system). The Ref-Syst was associated with a positive change in the caesarean rates in all the residence area strata. Right after this intervention the rates increased for the women living in cities with hospitals and those living in villages without a health facility. The trends changes were all significant, indicating an increase of the caesarean rate for the general population as well as all the subgroups, with the exception of women living in villages with a health centre, where the rate decreased.

## Discussion

The main finding of this study is that the Free-CSec policy affected the caesarean rate only for women living cities with a hospital and not for those living elsewhere, with the latter group accounting for 91% of our study population. This lack of significant impact for some subgroups was due to a previous increase in the caesarean rate related to implementation of the Ref-Syst. Seven years and 11 months after the introduction of free caesareans, the rate was 5.7% for women living in cities with district hospitals and 1% for women in villages with no health facility. Regrettably, this result was not entirely unexpected. In subsidising only the hospital costs associated with caesareans, this measure fully benefited women for whom these were the main expense, but only represented a partial exemption for women who had to incur additional costs. Cavallaro and al. [33] showed that in Sub Saharan Africa, caesarean delivery rates are low and even lower in rural areas and for the poorest households. For Mali, we report higher rates but our estimates are different (living births vs. deliveries) and our study period is more recent (2003-12 vs 1996–2006).

A review on Malian caesarean rates based on routine data showed an increase from 0.5% to 1.6% in the Kayes region between 2005 to 2009. Our figures for the same periods are 0.62% and 1.24%, but our study area covered only 5 of the 7 districts of the Kayes region [34].

In Senegal, an increase from 4.2% to 5.6% in the rate of facility-based caesarean deliveries in one year was reported, a positive impact despite problems in the implementation [35]. These rates are not directly comparable to those of our study; they would result in population estimates for caesarean rates of 1.7% and 2.5%. In one district of Burkina Faso, the implementation of a comprehensive intervention that significantly reduced costs led to a rise in the population caesarean rate from 1.9% in 2003 to 3.3% in 2006, with much higher rates in urban (4.6%) than in rural (0.8%) populations [5]. The rate increases in our study are higher than those reported in Senegal and Burkina Faso. This is not surprising since the observation period was longer and the starting rate was much lower, offering lots of room for growth due to significant unmet needs.

In several countries the costs of a caesarean represented 11% to 138% of the per-capita GNP and the exemption policies did not cover transportation costs [36]. These costs and other expenses incurred by patients and their families (food, accommodation) were estimated to represent 11% to 54% of the total costs of obstetrical complications in Benin [37]. In Mali a comparison of the wealth distribution of women delivering via caesarean section showed that women from the two wealthiest quintiles accounted for 58% of all the caesareans [38].

In terms of coverage of needs, the 5.7% caesarean rate for women living in cities with district hospitals meets the minimum threshold of 5% determined by WHO [10]. At this level in sub-Saharan Africa, most obstetrical complications, such as foetal-pelvic disproportion, abnormal presentations, total placenta praevia, or prevention of uterine rupture in cases of prolonged labour, are being treated [8], [9]. Conversely, when rates are around 1%, some caesareans that are absolutely medically indicated are not carried out, leading to serious complications for mothers and newborns, even to their deaths. This is unfortunately still the case for women living in villages without any healthcare facility in the Kayes region, despite Mali's free caesarean policy. Nevertheless, the separate analysis of the proportion of caesareans for AMI showed a decrease 3 years after the introduction of the Free-CSec policy, especially for women living in cities with a hospital. This is a major threat to the potential maternal benefit of the caesarean section.

This study's major strength is that it covered a period of approximately 10 years and was able to analyse the effects the Free-CSec policy while taking into account the effects of an earlier program for improving access to EmOC. Another strength is the quantity and quality of the data used to produce reliable numerators for estimates of population caesarean rates and to stratify the analyses using a variable for geographic and organisational accessibility, which is of great interest. The main potential weakness concerns the estimated denominators. The comparison of caesarean rates used for this study and direct population measures was done by using data from the 2006 Demographic and Health Survey (DHS) [18] which was the only representative population survey available and for similar zones and periods. The DHS data are representative at both the national and regional levels. For the Kayes region, the sample consisted of 1,686 women. This estimate took into account the anomalies noted by Holtz and Stanton [39] in determining population caesarean rates using the DHSs. The population caesarean rates obtained from the Mali DHS was 1.77% for the period from January 2003 to June 2005 and 1.24% according to the GESYRE. This difference is not statistically significant. Comparisons of sub periods led to the same conclusions.

Interrupted time series are considered to be among the most robust designs for observational studies to evaluate interventions [40] and they are included in the EPOC Group journals [41]. The main threat to the internal validity of interrupted time series is changes in the population over time (selection), in the methods for measuring outcomes or the emergence of other interventions or events that influence outcomes (history bias) [26]–[27]. During the study period, the population consisted of all caesarean cases registered in the database, and their inclusion in the database was based on strict criteria that did not change over time. We compared the age of the women (16 and less, 17–34, 35 and more) after and before the intervention and it did not changed (Χ^2^ = 1,07, p = 0.293). The method for measuring outcomes also did not vary over the period studied: the research team carried out all the visits to collect and validate the data in the GESYRE. Over the period 2003–2012, the two other dimensions of accessibility that may have contributed to improving access to caesareans were the implementation of the Ref-Syst and the service offer. With respect to the service offer, the number of points of service performing caesareans remained constant (five district hospitals), but the number of primary healthcare centres not performing caesareans increased from 94 to 167. We took into account different periods, so we were able to measure the effects of free caesareans while taking into consideration the effects inherent to the Ref-Syst. A “non-treatment” group should have improved our design [42] but such a group was not available for a so long time series.

## Conclusion

From 2003 to 2012, the caesarean rate increased from 0.25 to 1.5% in five districs of the Kayes Region. The Free-CSec policy adopted by the Mali government to improve access to obstetrical services had positive and significant effects only for women living in cities with a hospital of our study population. A previous intervention, the Ref-Syst, also induced a strong change in this trend. However, disparities related to location of residence are deepening. This gap has led to a paradoxical situation: for some segments of the population, seven years after the introduction of free caesareans the rates are still too low to have any impact on maternal mortality, whereas for others, the very serious needs that this intervention was intended to cover have been effectively addressed, even surpassed, to the point where further expansion could actually lead to damaging effects. As the proportion of caesareans for absolute maternal indications is decreasing, the effects of the Free-CSec policy on maternal health are also probably diminishing.

A lesson to be drawn from this evaluation is that a comprehensive intervention (Ref-Syst) addressing several barriers can produce positive long term and equitable effects. In contrast, a narrowly designed intervention (Free-CSec) produces positive effects for a limited part of the population and increases inequities.

The free caesarean policy is a measure that has improved access to this essential service, and it should be sustained. However, to improve its effectiveness and reduce inequities arising from its implementation, there needs to be more complete coverage of costs, in particular the costs of transportation and of accommodation for families living far from the points of service.

## Supporting Information

Figure S1 **Interrupted times series: after-before referral system.**
(TIF)

Figure S2 **Fitted lines after-before referral system.**
(TIFF)

Table S1 **Parameter estimates from the segmented regression model for the referral system.**
(DOCX)

Text S1 **Population and deliveries estimates.**
(DOCX)
