# Supplementary figures and images for: The Free Caesareans Policy in Low-Income Settings: An Interrupted Time Series Analysis in Mali (2003–2012)
Source: PLoS One. 2014 Aug 19;9(8):e105130. doi: 10.1371/journal.pone.0105130 (PMC4138145; doi:10.1371/journal.pone.0105130)

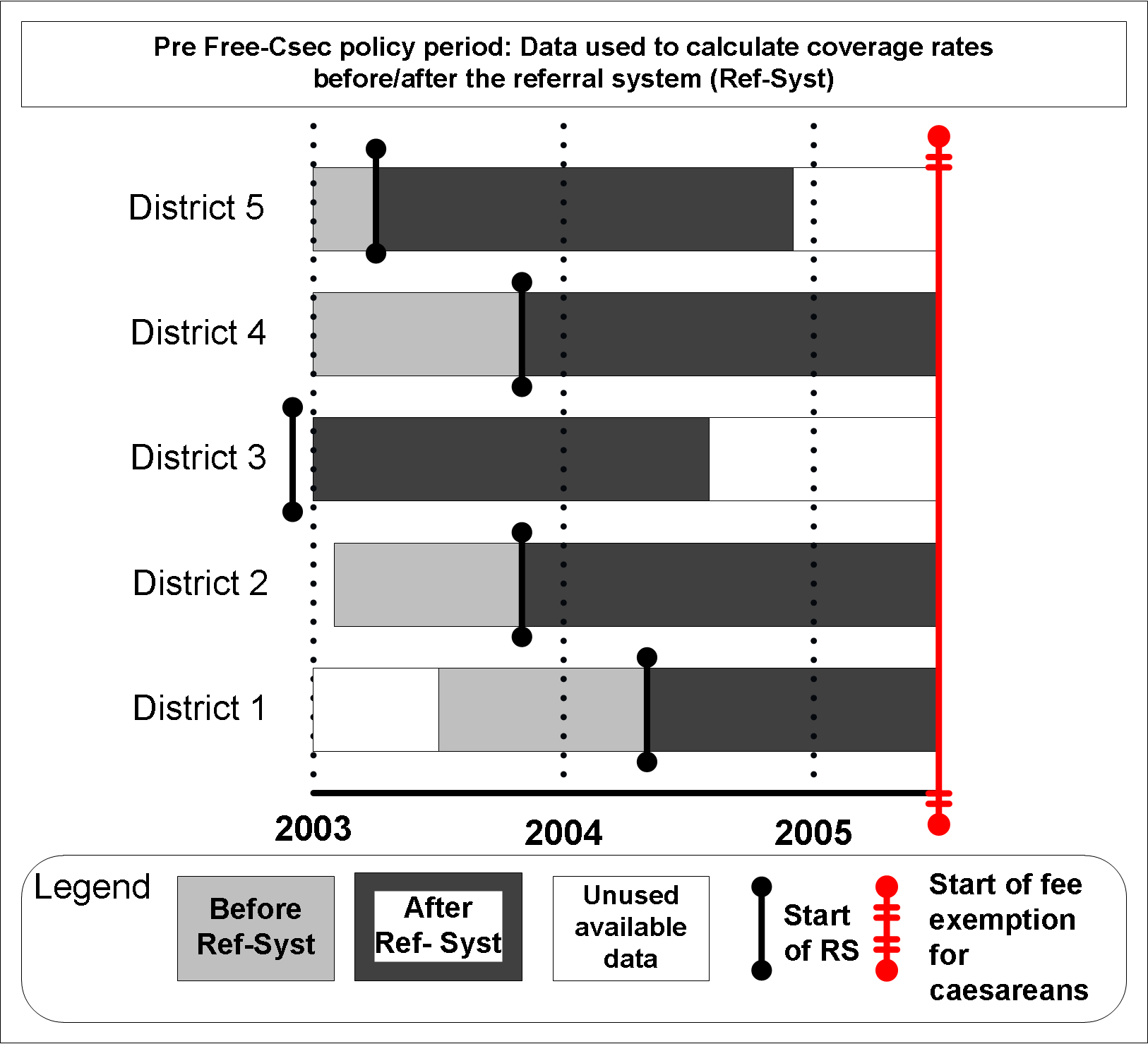

Supplement: Figure S1 — Interrupted times series: after-before referral system. (TIF) [file pone.0105130.s001.tif]
