# Supplementary material for: The Free Caesareans Policy in Low-Income Settings: An Interrupted Time Series Analysis in Mali (2003–2012)
Source: PLoS One. 2014 Aug 19;9(8):e105130. doi: 10.1371/journal.pone.0105130 (PMC4138145; doi:10.1371/journal.pone.0105130)
Supplement: Table S1 — Parameter estimates from the segmented regression model for the referral system. (DOCX) [file pone.0105130.s003.docx]

Pre fee exemptions for caesareans period: Parameter estimates from the segmented regression model for the Referral System

Intervention based on Newey-West method (10 months before and 20 months after)

| **Areas of residence** | **Coefficient** | **S.E.** | ***t*-statistic** | **p-value** | **95% CI** |
| --- | --- | --- | --- | --- | --- |
| **Villages with no healthcare facility** |  |  |  |  |  |
| *Fit test (Wald test)^b^* | *F=14.61; p<0.001* | | | | |
| Intercept (β0) | 0.281 | 0.037 | 7.59 | <0.001 | (0.21; 0.36) |
| Trend before the intervention (β1) | -0.017 | 0.006 | -2.70 | 0.012 | (-0.30; 0.00) |
| Change right after the intervention (β2) | 0.088 | 0.035 | 2.49 | 0.019**^CA^** | (0.02; 0.16) |
| Trend change after the intervention (β3) | 0.026 | 0.008 | 3.43 | 0.002**^CT^** | (0.01; 0.04) |
| Absolute effect (S.E) | 2.246 | 0.658 |  |  |  |
| **Villages with a healthcare centre** |  |  |  |  |  |
| *Fit test (Wald test)^b^* | *F=68.98; p<0.001* | | | | |
| Intercept (β0) | -0.018 | 0.042 | -0.42 | 0.679 | (-0.10; 0.07) |
| Trend before the intervention (β1) | 0.054 | 0.008 | 6.60 | <0.001 | (0.04; 0.07) |
| Change right after the intervention (β2) | -0.140 | 0.110 | -1.28 | 0.212 | (-0.37; 0.09) |
| Trend change after the intervention (β3) | -0.024 | 0.010 | -2.40 | 0.024**^CT^** | (-0.04; 0.00) |
| Absolute effect (S.E) | -2.132 | 0.808 |  |  |  |
| **Cities with a district hospital** |  |  |  |  |  |
| *Fit test (Wald test)^b^* | *F=9.13; p<0.001* | | | | |
| Intercept (β0) | 1.603 | 0.179 | 8.93 | <0.001 | (1.23; 1.97) |
| Trend before the intervention (β1) | -0.099 | 0.028 | -3.48 | 0.002 | (-0.16; -0.04) |
| Change right after the intervention (β2) | 0.813 | 0.258 | 3.15 | 0.004**^CA^** | (0.28; 1.34) |
| Trend change after the intervention (β3) | 0.109 | 0.030 | 3.59 | 0.001**^CT^** | (0.05; 0.17) |
| Absolute effect (S.E) | 9.860 | 20.123 |  |  |  |
| **Total** |  |  |  |  |  |
| *Fit test (Wald test)^b^* | *F=26.68; p<0.001* | | | | |
| Intercept (β0) | 0.327 | 0.016 | 19.89 | <0.001 | (0.29; 0.36) |
| Trend before the intervention (β1) | -0.009 | 0.003 | -3.26 | 0.003 | (-0.01; 0.00) |
| Change right after the intervention (β2) | 0.095 | 0.051 | 1.85 | 0.075 | (-0.01; 0.20) |
| Trend change after the intervention (β3) | 0.024 | 0.006 | 4.00 | <0.001**^CT^** | (0.01; 0.04) |
| Absolute effect (S.E) | 2.087 | 0.618 |  |  |  |

^a^ Wald test of simple and composite linear hypotheses

**^CA^** Significant change right after the intervention

**^CT^** Significant change in trend after the intervention
