# Supplementary material for: The Free Caesareans Policy in Low-Income Settings: An Interrupted Time Series Analysis in Mali (2003–2012)
Source: PLoS One. 2014 Aug 19;9(8):e105130. doi: 10.1371/journal.pone.0105130 (PMC4138145; doi:10.1371/journal.pone.0105130)
Supplement: Text S1 — Population and deliveries estimates. (DOCX) [file pone.0105130.s004.docx]

**Text S1: Population and deliveries estimates**

Population estimates by district and communes

The 1998 and 2009 censuses^[[1]](#footnote-1)^ provide direct measures of the population by district and commune^[[2]](#footnote-2)^. To estimate the population between 1998 and 2009 a specific growth rate was computed for each of the 69 communes. Population changes showed huge differences between the communes: average growth was 3.45% per year with a maximum of 9.6% and a minimum of – 3.3%.

The same rates were applied to estimate the population between 2010 and 2012.

Number of deliveries estimates

The expected number of deliveries was estimated by multiplying the population estimates by the annual crude birth rate in Mali that varied from 49.166 to 45.867 per 1000 between 1998 and 2011. For 2012, the decrease rate observed between (2008 and 2011 (5.4 per 1000) was applied.

Numbers of caesareans

All obstetrical emergencies are systematically registered in all the hospitals of the region in a computerised database (GESYRE), including both planned and emergency caesareans. In the study area there are no private institutions carrying out caesareans. The location of residence before delivery is registered for each patient. Every six months, the research team verified the data in each district with respect to the criteria for inclusion in the database, the exhaustiveness of the cases to be included, and missing data. The district health teams use these data to produce activity statistics for several maternal and child health programs as well as to analyse trends and the effects of the Ref-Syst at the local level. The data are captured using Microsoft Access^©^.

Caesarean rates

To obtain the most reliable population estimates possible, only residents of a given district or zone were reported in that population of origin (for example, a resident from the main city of District A who had a caesarean in District B was counted in the numerator of District A’s population rate).

1. Ministère de l’Économie et des Finances, Direction Nationale de la Statistique et de l’Informatique. Recensement général de la population et de l’habitat, Avril 1998. Principaux Résultats. Bamako : INSTAT; 2001.

   Ministère de l’Économie et des Finances, Institut National de la Statistique, Bureau central de recensement. 4^ème^ recensement général de la population et de l’habitat du Mali (RGPH). Résultats Définitifs. Tome 1 : Série Démographique. Bamako : INSTAT; 2011. [↑](#footnote-ref-1)
2. They are two kind of commune: urban and rural. An urban commune is a city while a rural commune is a group of villages or hamlets. [↑](#footnote-ref-2)
